# 2. The Data Librarian: introducing the Data Librarian

**DOI:** 10.1155/S1463924697000242

**Published:** 1997

**Authors:** Joe Liscouski

**Affiliations:** LASF PO Box 38 Groton MA O1450 USA

## Abstract

This paper provides some initial considerations into the design and function of the Data Librarian. The first part (Liscouski, J., 1997, Journal of Automatic Chemistry, 19, 193-197) described the need for the Librarian.


Journal of Automatic Chemistry, Vol. 19, No. 6 (November–December 1997) pp. 199–204

### 2. The Data Librarian: introducing the Data Librarian

**Joe Liscouski**

*LASF, PO Box 38, Groton, MA01450, USA*

*This paper provides some initial considerations into the design and function of the Data Librarian. The first part* (Liscouski, J., 1997, *Journal of Automatic Chemistry*, **19**, 193–197) *described the need for the Librarian.*

The Data Librarian addresses the following problems:

(1) The management of large numbers of data files.
(2) Searching data files for information that meets user defined criteria (type of contents, creation date, who created it, intended application, etc.).
(3) Security for, and access to, data in a shared multi-user environment, including the client/server.

Consider the following problem in an office environment: you work with a large and growing number of documents, and at any point in time you may need access to any documents created by you or anyone else on a particular topic. The documents may be text, letters, contracts, photographs, drawings, etc. How do you manage them and guarantee access to the material you need?

Office workers using computer systems have that problem on a daily basis, compounded by the limitations of current computing technology. Suppose each document had to be stored in an envelope and the only identification on the envelope were the date of creation and an 11-character identification code. Since several people in the office need access to all documents, they are all stored in the same filing system, with each worker responsible for assigning their identification code. In case of duplicate codes, the contents of the earlier material may be lost. Computer systems force their users to identify documents—files—by a character code called the file name. On DOS and Windows systems you are limited to an 8-character name with a three-character extension. Macintosh users can use 31 characters. If two files are placed in the same storage area at the same time, the earlier file can be lost if care isn't taken. How long would it take before chaos reigned? How hard would it be to find all documents relating to a particular project between two dates for all workers?

Automated systems in laboratories can generate hundreds of files per day, all of which have to be maintained in a secure environment to meet regulatory and legal requirements. The purpose of the Data Librarian is to provide a means of managing, storing, retrieving, and searching large numbers of files from many sources in a single system. Laboratory managers are complaining about the amount of data available and their inability to find and use what is needed. The Data Librarian solves these problems.

### What is the Data Librarian?

The Librarian is a software package designed to manage data files created by laboratory instrumentation. Data format standards are being developed to permit laboratory personnel to export data from instrumental analysis systems. This is going to result in the creation of a large number of files, with the potential for data loss, mismatching file names and what is expected to be in them.

The Data Librarian will provide a means of storing, retrieving, searching and recording access to laboratory data. In addition it provides:

(1) Long-term archiving of laboratory data—an increasingly significant consideration for regulated industries and product liability cases.
(2) A means of preventing data loss.
(3) The basis for developing a system of third party analysis and reporting packages.
(4) A required function in the development of integrated laboratory systems.
(5) A significant step in solving the instrument-to-LIMS data connection problem.

Within the information flow diagram developed by the Laboratory Automation Standards Foundation (LASF), the Librarian package occupies a pivotal position (figure 1)—the full details of the LASF model are covered elsewhere (*Laboratory and Scientific Computing: A Strategic Approach*, J. Liscouski, John Wiley, 1994). Laboratory data, collected and formatted by instrument data systems, are exported in files meeting industry standard data structures (figure 1).

File names and locations are decided by the end-user. Without the Librarian, these files would be distributed in directories established by the user on one or more computers, each having its own problems of backup, security, and risk of data loss. A very real potential exists for re-use of file names which would cause the loss of data. The contents of files and their names would have to be managed by each user. Unless programming was developed by the system users, there would not be any mechanism for searching data, aside from a brute-force review of the manually managed file log.

The Librarian offers an automatic method of storing and retrieving instrumental data. It would manage the files, provide for searches, distribute the data across media to optimize access and reduce storage cost. Programs, using built-in accesss controls, can access any data for analysis and reporting. The system would maintain logs of data access, audit trails and parent–child relationships between files; facilities that are needed to meet regulatory requirements (Good Laboratory Practices [FDA, EPA], current Good Manufacturing Practices [FDA], Good Automated Laboratory Practices [EPA], and ISO

0142-0453/97 $12·00 © 1997 Taylor & Francis Ltd

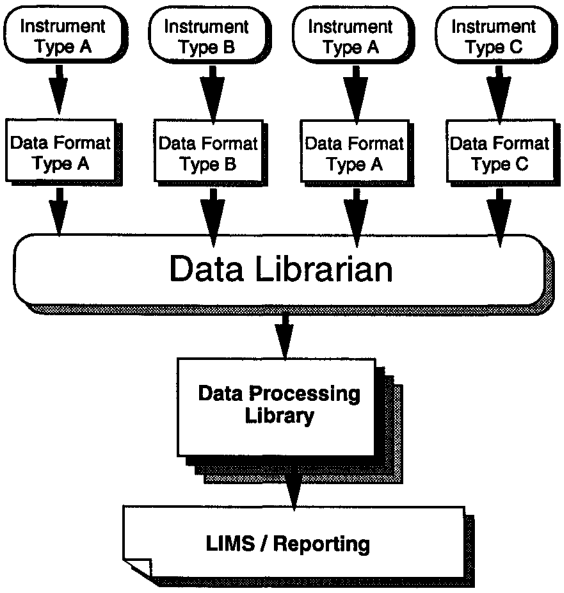

*Figure 1. Information flow in a laboratory.*

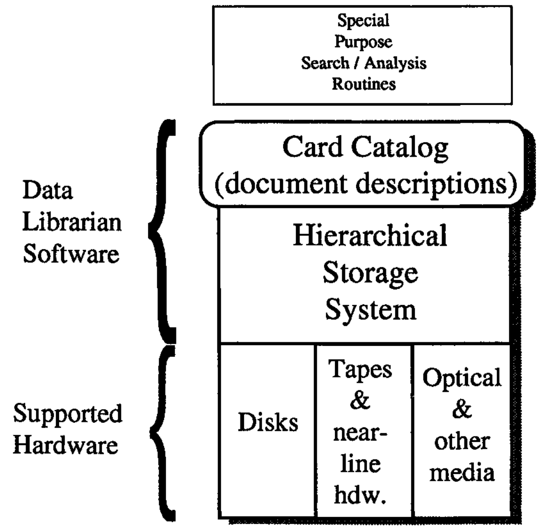

*Figure 2. Basic structure of the Data Librarian.*

9000). These logs would also provide a chain of custody required to support claims in product liability cases.

Once the Librarian is in place four things will happen:

(*a*) Laboratories will be in a better position to manage and use valuable data—solving an increasingly acute need.
(*b*) It will be possible to develop libraries of data analysis and reporting packages—developers and researchers can concentrate on analysis procedures without having to create an entire acqusition–storage–analysis–reporting system.
(*c*) It will be practical to transfer data automatically into LIMS systems without the need for a customized data transfer package—reducing the cost of implementing laboratory automation systems.
(*d*) Validating laboratory systems to meet regulatory requirments will be simplified—reducing management and system costs.

### Comparison between the Data Librarian and LIMS

Information management has been part of the laboratory software environment since 1982 in the form of Laboratory Information Management Systems (LIMS). LIMS are designed to manage small quantities of data and the administrative information needed to run a laboratory (what work has to be done, what has been recently completed, prioritizing workloads, etc.). Effective data management, that is handling the large volumes of data coming from instruments that are reduced to a few descriptive values, has not been adequately addressed. Table 1 compares the Librarian to a LIMS.

Figure 2 shows the basic structure of the librarian. The heart of the system is the Card Catalogue which provides all of the descriptive information about each file stored. This is supported by a Hierarchical Storage System (HSS) that directly manages the hardware, file allocation, and migration between media.

There are existing HSS from several vendors—they may be considered as possible components of the Librarian. All of them treat files as discrete entities, with the supported hardware acting as one large storage device. They do not provide the facilities of the Card Catologue—a key item for resolving file name conflicts and searching files. In some cases, the HSS is provided by the hardware vendor as a means of selling the underlying hardware, just as computor vendors once offered operating systems as a means of getting customers to buy their hardware. The parallel between operating systems and HSS is strong: in both cases the vendors who have concentrated on hardware sales are beginning to appreciate the impact of software on applications design and systems implementation. Today, operating systems and applications software drive hardware sales. File and information management systems will drive the storage market and have a major impact on the design and implementation of client/server applications design.

Vendors such as Oracle, Sybase, Informix, and others provide database development products that are not competative with the Data Librarian. These products may, pending an examination of security issues, provide a basis for the Card Catalogue.

### Initial system considerations

### *Scalability and growth paths*

The system needs to be scalable—able to manage data collections on PCs (including the Macintosh), UNIX systems and mainframes. A clear growth path from PC-sized systems to mainframes needs to be established, so that users that outgrow PCs can move into larger systems.

*Table 1. Features of the Data Librarian.*

| *The Data Librarian is:* | *The Data Librarian is not:* | *Comments* |
|---|---|---|
| A database system for managing instrument data in standards compliant format. | A substitute or replacement for a LIMS. | The primary purpose of a LIMS is to manage sample information and the conduct of work in a testing laboratory. The two packages are intended to be complementary and not competitive. The Data Librarian is an alternative to extending commercial LIMS to accept instrument readings. It will also find utility in laboratories whose basis is not samples but organizing information by other criteria. |
| A framework that third-party vendors can use to add features needed by a particular laboratory or industry. | A framework that third-party vendors can use to add features needed by a particular laboratory or industry. | The Librarian is not intended to directly provide data analysis and reporting, rather it is a framework for the thorough management of data with a standard, published, programming interface that third-party packages can use to access and work with data. This approach will encourage companies to add functionality via plug-in modules (some that may be customized for particular industries, such as environmental analysis and reporting) rather than creating complete competitive systems. This approach will lead to using the Librarian as a base, but still give vendors the differentiation they require in a competitive market. |
| The basis for integrating diverse analytical techniques, analysis procedures, reporting, and archiving in a single system, with standardized links to complementary LIMS systems. | A threat to existing data system vendors. | The Librarian is a recipient of data acquired by a data station. In small laboratories with one instrument technique the Librarian would serve a long-term archiving function. In larger laboratories it would provide the basis for the extended analysis of materials by multiple analytical techniques, in addition to its long-term storage function. |

*Table 2. Advantages of the Data Librarian.*

| *Features* | *Benefits* |
|---|---|
| Incorporates a hierarchical storage management system. | Rather than having to manage files stored on individual disks, tapes and other media, the system can be viewed as one very large device. The components of that device can be varied to optimize against speed of access, storage cost, and other parameters. New technologies can be incorporated without any negative impact on the system. |
| Incorporates a 'Card Catalogue'. | Permits the user to search for data files efficiently using pre- and user-defined keys. This means that data can be found when needed, reducing the need to duplicate work because the initial results could not be found or verified, and will help meet regulatory and legal requirments for data management. Data becomes a usable corporate asset, rather than a management nightmare. |
| Data in managed in standardized formats. | Developers on third-party analysis packages can develop software for specific applications without having to build otherwise extraneous software and hardware—this should stimulate the development of applications software, giving the user more choices of tools for getting work done. |
| Provides a basis for integrating laboratory data and LIMS. | Reduces the cost of laboratory automation systems design and implementation—today this is a major stumbling block, one that frequently leads to failure in the initial implementation attempts. |

### *Database design*

The database design should follow the IEEE Mass Storage System Reference Model.

The intent of this product is not to create a system that manages data, but one that manages data files—this distinction is significant to system design. The Librarian does not have to incorporate the laboratories data into a database, but neeeds to manages the location of data files, avoiding filename conflicts in the process. Some information about the data files needs to be associated with the master file directory to make searching efficient. User applications need access to the detailed data through the files. (People manage books without knowing the details of the contents . . . once you've found a book that interests you, you can look for the details; similarly, the Librarian manages books, not the contents of books.)

Part of the Librarian's function will be the ability to read the data files. Reading administrative information from the files, rather than having the user type it in, will provide for file verification and ease of use. This means that 'filters' will have to be available in a directory that will tell you how to read the data files. The list of filters would be updated without interrupting the Librarian.

The Librarian needs to operate in nonstop mode, and be able to recover from less-than-graceful shut-downs (power failures, etc.).

Each data file would have an associated history file that travels with it as it migrates from one media to another. File usage history (chain of custody, etc.) is important and may be necessary to satisfy regulatory agencies and demonstrate data integrity for legal usage.

As the library of files grows, media management becomes important, and the system has to manage the migration of files to and from tape, disks, optical media, RAID systems, etc. Unitree (formerly from General Atomics, now part of Open Vision) is one software system that does this. It is based on software produced at Lawrence Livermore National Laboratories—LLNL and other American labs. NASA may have software in the public domain or available as part of defence conversion projects that could assist in producing the Librarian.

The Librarian should not be designed with traditional commercial database systems unless it can be demonstrated that there is adequate security to prevent tampering with the database. These systems provide a number of tools for programmers to use to access their contents. These tools can be used to subvert audit trails and history files, and that should not be permitted. Any information within the data system should be accessible to user in printed or machine readable form—we are not trying to control their data, just manage it—but we should take all steps to prevent tampering.

Data entry, search, and requests for data should be possible through an interactive mode or program access. Under program access it is conceivable that a user may point to a directory and say 'enter all of the files in that directory into the system'.

*Security*

Assume the worst. The FDA has recently produced a specification for the electronic identification of individuals. The software should incorporate those specifications.

### Librarian functions

The basic functional model of the Data Librarian is the circulation desk of a large public library, with the following additions: all transactions must be recorded, people who 'borrow' a book do not get the data file but a copy of it, putting a file back does not replace the original file, but creates a new entry with a pointer to its predecessor. *Note:* files do not have to be returned if no changes were made. Basic functions should include:

(1) Initial submission of a file—either manually or through program access to a port. A batch file submission should be possible, an initial query to the user should provide all necessary, repetitive or predictable data (counters, prefixes, etc.).
(2) Request of copy of a file, or a set of files, given a search criteria.
(3) Return a copy of a file—returning a copy of a file should always be treated as a new submission with the addition of pointers to the original file.
(4) Removing files—this is an interesting problem. In regulated environments, files should not be deleted as a normal practice. If they are, a record of who deleted them, when, why, and who authorized the deletion needs to be kept. In some systems—non-regulated research for example—deletion of old or pointless data is normal practice. Regulatory support should not be able to be turned off or on, or overridden. It is either there or it isn't.
(5) Catalogue search—obtain a list of files with user supplied search criteria. There may be two modes: searching based on criteria in the master file (should be fast), and, searching based on the contents of data elements within data files or history files (very slow since some data will be off-line).
(6) Maintenance functions—adding or updating filters, etc.

*Initial design/functional considerations for the Data Librarian*

Figure 3 shows a breakdown of the key components of the Data Librarian System (DLS). The primary designs goals are:

(1) Simplicity.
(2) Ease of implementation—thus a higher likelihood of successful implementation.
(3) Modularity—to ease implementation (increase the potential for parallel development paths), flexibility.
(4) A product that works.

The DLS is a client-server system. Human/instrument data system interactions take place at the client, automatically providing a distributed processing environment, and communicates with the server via messages (requests for action). That communication takes place over a public network provided by the users company. Clients may be any type of processor system including Macintosh, PC, Unix, etc.

The Data Librarian resides on a dedicated server with its own mass storage and backup facilities. The implementation will be based on Windows NT or Unix. Within the server are four basic modules:

(*a*) A Monitor—its function is to provide system security against viruses and any unauthorized activity. It will not permit, for example, anyone to load a program onto the server and execute it.
(*b*) The Transaction Broker—all communications with the user world is done through this entity. It captures all messages, determines what action has to be taken, and then carries out that action. Possible actions include: copying a file to a client, carrying out a search of the card catalogue, entering a file into the system, etc. An application programming interface (API) needs to be developed to allow client applications to access the Transaction Broker. That API needs to include a DLS_OPEN/DLS_CLOSE function that can be used in place of a normal program file open/close operation, thus treating the DLS as a large storage device.

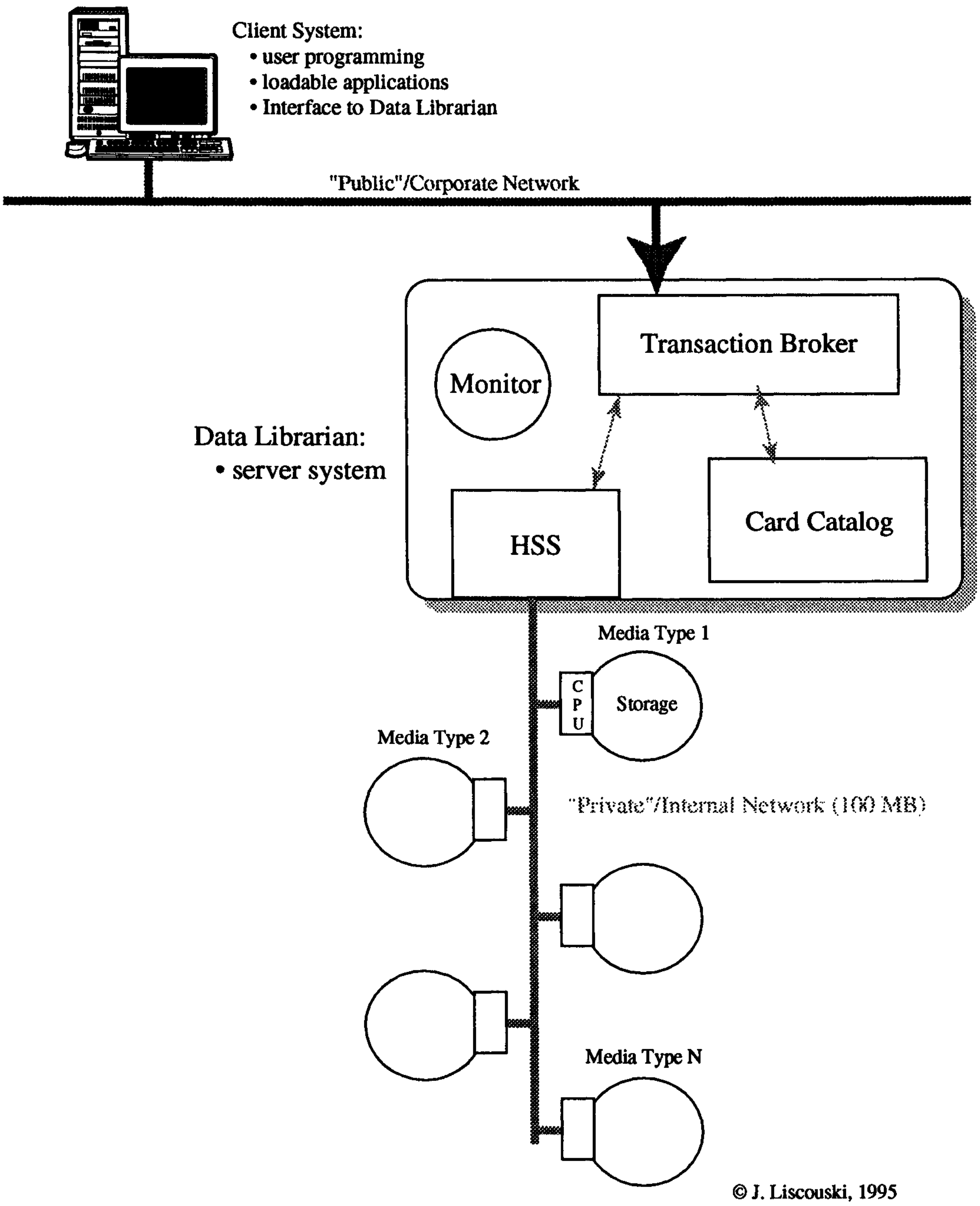

*Figure 3. Key components of the Data Librarian System (DLS).*

(*c*) The Card Catalogue—this is a basic database application that contains searchable information (attributes) on all files within the librarians structure. It is logically independent of other modules so that its implementation can be optimized without compromising other data structures. Among the attributes a file can have are: its registration string, original file ID, source, data/time stamp, data type, etc. as well as user definable attributes.

(*d*) The Hierarchical Storage System (HSS)—this is a classical HSS based on the IEEE Mass Storage System Reference Model. It is, from the standpoint of everything else in the system, a single, very large storage device. It is logically independent from the rest of the structure to allow for flexibility. It may be a third party commercial product (based on vendor alliances/user requirements) or an internally developed sub-system that could be a separate product offering. If multiple sources of the HSS are considered, the Transaction Broker would have to be able to deal with all of them.

*Hierarchical Storage System (HSS)*

The HSS has to be able to cope with several issues: it has to be able to be expanded as the users requirments grow; that expansion has to include new media types, as well as more of a given media; it has to operate in as much of a nonstop mode as possible with current technology.

In order to meet these requirements, an independently developed HSS should be based on a separate network

(100 MB/s minimum speed) that is not accessible by any user (logically and physically isolated from the public network). The network would act as the device bus on most computers. Each device would be managed by a CPU. Adding a new device would be a matter of adding the CPU/storage system to the network, initializing it, and then notifying the HSS that it was available, the rules for use, capacity, etc. and then, using it. This approach would allow new media types to be added as the technology developed, transfer files from older, obsolete media, and keep the system technically current.

*Transaction Broker*

Among the functions that this system would provide through client programs are:

(i) Individual, manual, file registration—a user notifies the system that they want to register a file, providing the attribute definitions through a GUI, and then at the completion of the action, being notified of the success/failure of the operation and the registration string.
(ii) Bulk, batch file registration—the DLS is told where to get the attributes for each file, and the source of the files. They are registered/transferred and a log is provided to the user for each transaction.
(iii) File deletion—not supported, violates regulatory requirements.
(iv) Updating/revising file attributes—adding modifying attributes with an adult trail.
(v) Request for copies of one or more files—file can be requested by registration number, file name, or search criteria. If only one file is requested, the user can specify a file name in addition to the destination location. If multiple files are requested, the files will be copied with the names matching the registration string (needed to avoid confusion). The user can change these names later through normal OS operations.
(vi) Support for a programmer interface.

